# Health Insurance Ownership and Quality of Computed Tomography Requests: Experience from a Peripheral Referral Hospital in Cameroon

**DOI:** 10.1155/2021/9959114

**Published:** 2021-06-21

**Authors:** Joshua Tambe, Yannick Onana, Sylviane Dongmo, Georges Nguefack-Tsague, Pierre Ongolo-Zogo

**Affiliations:** ^1^Division of Radiology, Department of Internal Medicine and Pediatrics, University of Buea, Buea, Cameroon; ^2^Department of Public Health, Centre for Research and Training in Graduate Studies in Life, Health and Environmental Sciences, The University of Yaoundé I, Yaoundé, Cameroon; ^3^Department of Clinical Sciences, University of Ngaoundéré, Ngaoundéré, Cameroon; ^4^Biostatistics Unit, Department of Public Health, The University of Yaoundé I, Yaoundé, Cameroon; ^5^Department of Radiology and Radiation Oncology, The University of Yaoundé I, Yaoundé, Cameroon

## Abstract

**Background:**

Health insurance ownership facilitates access and minimizes financial hardship after utilization of healthcare services such as computed tomography (CT). Understanding the rational utilization of CT by people with health insurance can help optimize the scheme and provide baseline information for a national universal health coverage program.

**Objective:**

To assess the relationship between health insurance ownership and the appropriateness of requests for CT in a peripheral referral hospital in Cameroon.

**Methods:**

A survey of CT users was conducted during which information on health insurance ownership was collected and the request forms for CT assessed for appropriateness using the American College of Radiologists (ACR) Appropriateness Criteria®.

**Results:**

We consecutively enrolled 372 participants of which 167 (45%) were females. The median age (range) was 52 (18–92) years. Thirty-eight out of 370 participants reported having health insurance (10.3%; 95% confidence interval (CI): 7.2%–13.4%). Twenty-nine out of 352 CT scan requests (8.2%; 95% CI: 5.3–11.0) were judged to be “inappropriate.” The proportion of inappropriate scan requests was higher amongst people with health insurance compared to those without health insurance (18.4% vs. 7.0%; *χ*^2^ = 5.8; *p*=0.02). In the logistic regression analysis, health insurance ownership was associated to the appropriateness of CT requests in the univariate analysis only (OR = 0.33; 95% CI: 0.13–0.84; *p*=0.020).

**Conclusions:**

Inappropriate requests for CT were low but nevertheless associated to health insurance ownership. The continuous sensitization and training of physicians would help minimize potential wasteful utilization of resources.

## 1. Introduction

The continuous development of advanced healthcare technology such as computed tomography (CT) has led to an increase in the cost of healthcare [1]. The utilization of multislice and extreme detector CT equipment with a very broad range of clinical applications has especially led to the price hike of CT procedures [1]. A high cost for healthcare services has the potential to limit access to care as people in need of the services may not be able to afford for them [2, 3]. Affordability of multislice CT technology is therefore perceived to be a barrier to its utilization, especially in settings without any financial protection for health. However, the affordability barrier to CT utilization can be minimized by the implementation of financial protection schemes [3]. Health insurance ownership is therefore expected to protect CT users from impoverishment and financial hardship that could arise from its utilization [3].

In Cameroon, CT scans have been installed in recent years in some peripheral referral hospitals around the country so as to improve upon the geographical accessibility of CT technology. The improved geographical accessibility is expected to reduce the time to obtain CT and also minimize inconveniences linked to long-distance travel to other towns. These peripheral health facilities which have been focusing on primary and secondary care now have to incorporate CT utilization during routine clinical practice with the potential for better health outcomes for patients. Nevertheless, CT, being a referral technology with a high radiation exposure, requires rational cost-effective utilization [1, 4–6]. According to a national survey, 96 to 98% of the general population do not have health insurance [7]. Whilst awaiting the implementation of a national universal health coverage program, available financial protection schemes for health include private insurance policies and community-based mutual and employer-provided insurance schemes.

Given the improved access to healthcare services conferred by health insurance ownership [8, 9], it is important to explore the rational utilization of services such as CT by people who have subscribed to health insurance policies. The information generated can help restructure available private health insurance policies and also shape the planned implementation of a public national universal health coverage scheme. There is paucity of information on the utilization of CT by people with health insurance. A pilot study reported an association between inappropriate CT requests and health insurance ownership in an urban sub-Saharan context [10]. Further studies to ascertain this relationship from different socioeconomic and geographical contexts are important so as to provide empirical evidence. In this study, we explore this relationship in a peripheral intermediate-level referral hospital in Cameroon.

## 2. Materials and Methods

A cross-sectional survey of CT users was conducted. This study was approved by the institutional ethics committee of the University of Yaoundé 1 (108/UY1/FMSB/VDRC/CSD). Administrative authorization for the study was also obtained from the South-West Regional Delegation for Public Health (R11/MINSANTE/SWR/RDPH/82/786). The study was conducted at the Medical Imaging Centre of Regional Hospital Limbe. Regional Hospital Limbe is an intermediate-level referral hospital in the Southwest Region of Cameroon with the capacity of 200 beds. Designated as a Category 3 referral health facility in the health system pyramid of Cameroon where the categories range from 1 (tertiary care) to 7 (primary care), its main role is to provide secondary care. The geographic location of this health facility is peripheral with respect to the country's political capital Yaoundé. In recent years, Regional Hospital Limbe has benefitted from the installation of a 16-slice CT scanner in an effort by the government to improve on access to health technology.

### 2.1. Participants

CT users aged 18 years and above who consented to participate in the study were consecutively enrolled between March 2018 and February 2019. Informed consent was written and was obtained either from the patient or the caregiver.

**Figure 1:**
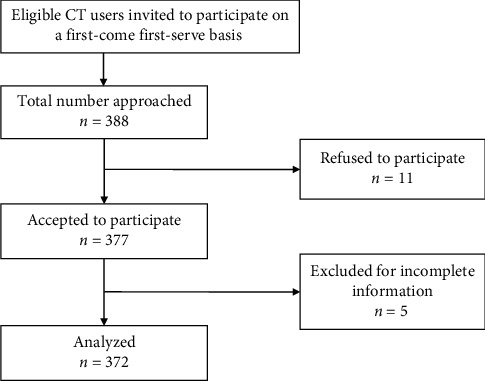
Participant selection flowchart.

Sample size estimation was done using Cochran's method for surveys with the appropriateness of requests for CT (expressed as a categorical binary variable) being the primary outcome [11]. Given an alpha level of 0.05, a 5% error margin, and a population variance of 0.25, the calculated sample size was 385 participants. Informed by a pilot survey [10], we expected data collection to last for 12 months, period during which an expected 1614 CT scans would be performed as anticipated from the hospital records. The estimated sample size of 385 exceeded 5% of this population, and the Cochran correction formula was applied [11] to give a minimum return sample size of 310 participants. Anticipating a nonresponse rate of 20% [10], a total of 388 potential participants were invited to participate in the study, and enrolment ended when a sufficient sample size was attained.  Figure 1 summarizes participant selection.

### 2.2. Data Collection

Data were collected from March 2018 to February 2019 using standardized forms. There was content validation of the included items on the data forms and pretesting through a pilot survey [10]. Data were collected on age, sex, educational achievement, marital status, occupation, socioeconomic status, health insurance ownership, and type of health insurance. Further information was gathered on the clinical indications for CT, anatomic region, and qualification of the referring healthcare provider. The appropriateness of CT requests was ascertained using the American College of Radiologists (ACR) Appropriateness Criteria® [12], which are guidelines that have been developed in collaboration with specialists from various domains with an aim to help referring healthcare providers to request for the best available imaging modality for specific clinical scenarios. During this study, the request forms for CT were used to determine appropriateness, and the final categorization was consensual between two radiologists (JT and POZ). CT request forms without any clinical indication were excluded from the study, whilst those with insufficient information were ignored from the appropriateness analysis. A research assistant collected all the data under the supervision of the principal investigator (JT).

### 2.3. Data Analysis

The data forms were transcribed onto a Microsoft Excel^®^ spreadsheet and analyzed using Stata^®^ 12 (StataCorp, Texas, USA). Continuous variables were summarized using the mean and standard deviation or median and range as appropriate. Categorical variables were summarized using frequencies, percentages, and 95% confidence intervals (CIs). Chi-squared tests were performed to compare proportions of inappropriate CT requests among categories of health insurance ownership. Univariate and multivariable logistic regression techniques were used to determine if any factors were associated with the appropriateness of requests for CT. For the multivariable modelling, covariates were entered as a block and included age, sex, educational achievement, socioeconomic status, health insurance ownership, and the qualification of the referring healthcare provider. Statistical tests were two tailed, and *p* values less than 0.05 were considered statistically significant. Model fit was assessed using the *R*^2^ statistic. The data were presented using tables.

## 3. Results

### 3.1. Participant Characteristics

Three hundred and seventy-two participants were surveyed of which 167 (45%) were females. The median age (range) was 52 (18–92) years. The demographic and socioprofessional characteristics of the participants are presented in Table 1. Thirty-eight out of 370 participants reported having health insurance (10.3%; 95% confidence interval (CI): 7.2%–13.4%). The reported health insurance types are presented in Table 2.

### 3.2. Appropriateness of CT Requests

Three hundred and fifty-two CT requests could be categorized for appropriateness. Of these, 29 (8.2%; 95% CI = 5.3–11.0) were judged to be “inappropriate.” Table 3 shows the categorization of CT appropriateness based on health insurance ownership.

The proportion of inappropriate scan requests was higher amongst people with health insurance compared to those without health insurance (18.4% vs. 7.0%; *χ*^2^= 5.8; *p*=0.02).  Table 4 summarizes the relationship between health insurance ownership and CT request appropriateness.

In the logistic regression modelling, health insurance ownership was associated with the appropriateness of CT requests in the univariate analysis only (OR = 0.33; 95% CI: 0.13–0.84; *p*=0.020). However, educational achievement beyond ordinary level was associated with the appropriateness of CT requests both in the univariate (OR = 0.42; 95% CI: 0.18–0.97; *p*=0.043) and multivariable analysis (aOR = 0.35; 95% CI: 0.13–0.91; *p*=0.032). The results of the logistic regression analysis are presented in Table 5.

## 4. Discussion

From the findings of this study, an estimated 10% of the respondents reportedly had health insurance ownership. This is higher than the 2–4% health insurance ownership of the general population following a national survey [7]. This difference could be explained by the fact that this survey focused on a subset of the population utilizing a particular health service and so not representative of the general population. The purchase of healthcare in Cameroon is essentially through direct out-of-pocket (OOP) payments. OOP payments lead to unequal access to care, whilst health insurance ownership minimizes access inequities [13–17]. Many governments around the world seek to improve access to care for its population through the implementation of a universal health coverage (UHC) scheme [3]. The benefits of UHC cannot be overemphasized, which is the reason why the government of Cameroon has embarked upon the creation of such a scheme in the near future. The anticipation of a UHC scheme motivated the assessment of the rational utilization of CT by people with existing insurance schemes.

The findings of this study support the fact that health insurance ownership confers better access to CT and also potentiates the likelihood of inappropriately using CT as health insurance ownership was independently associated to inappropriate requests for CT. It is likely that people with insurance may have some compulsion to access some health services even when these are not indicated just because they have subscribed to and contribute for such schemes. This may put pressure on healthcare providers to request for CT as they may also fear any potential litigation should they fail to request, and a serious condition is later detected.

Educational achievement beyond ordinary levels was also associated with inappropriate CT requests. Most of the insured participants had an insurance premium provided by the employer, and it is likely that a minimum level of education is required to get employed with a formal contract of employment. Furthermore, people with higher educational qualifications may be more demanding when accessing healthcare services with more pressure to obtain some services even when these might not be needed.

Inappropriate utilization of a health technology such as CT for whatever reason has to be given a serious consideration given that CT is associated to high exposure to ionizing radiation with the potential of radiation-induced cancers [4, 5, 18–21]. The cost of CT is also considerable irrespective of whether payments are made OOP or through insurance [1], and wasteful utilization will strain the pooled resources that have been made available for the scheme [3]. If CT utilization by a smaller population with health insurance shows inappropriate utilization patterns, then it is likely that this effect might be multiplied with the extension of health insurance to a wider population.

Geographic accessibility is fundamental to accessing healthcare services [22, 23]. The installation of CT in peripheral health facilities in cities distant from the capital cities ensures the decentralization of health technology. Patients no longer commute for considerable distances to access some health technologies as was the case, and utilization has all the chances of being timely too. Enhanced geographic access to health technology therefore has the potential to improve the quality of care and health outcomes. Following the introduction of health technology such as CT to peripheral health facilities, health practitioners have to incorporate its use during routine clinical practice. This may be simple for some clear-cut clinical indications and trivial for others. Healthcare practitioners therefore have to step up knowledge usually through refresher courses, continuous medical education seminars, and personal studies to be abreast with the clinical use of health technology.

Strategies to curb inappropriate utilization must therefore be envisaged while the government plans to implement a universal health coverage scheme. Firstly, given the absence of regulations as to who is qualified to request for imaging studies in the study setting, we suggest that CT requests should be approved only when prescribed by duly trained healthcare professionals. Also, consultations with radiologists should be encouraged if uncertain about the role of CT as more cost-effective imaging alternatives might be readily available [24]. Furthermore, continuous training and sensitization, the use of guidelines, and imaging decision support by referring physicians should be encouraged [25, 26]. Finally, a tracking system for all requested CT studies with regular reviews to assess prescription trends and relevance will provide useful feedback on utilization.

CT utilization and health insurance ownership have not been given much attention in the medical literature. Bellolio et al. reported an increase in CT utilization among commercially insured patients but did not assess the appropriateness of CT utilization [27]. Becker et al. also reported inappropriate utilization of both CT and magnetic resonance imaging without any assessment with respect to health insurance ownership [28]. This paper focused on the specific relationship between the health insurance ownership and the appropriateness of CT utilization, providing opportunities for scaling up the use of CT by people with health insurance in resource-poor settings.

### 4.1. Limitations

As limitations to this study, some CT scan requests could not be categorized for appropriateness due to insufficient clinical information on the forms. Also, reporting bias could have influenced the findings of this study.

## 5. Conclusions

The findings of this study support the fact that health insurance ownership, despite the proven benefits in minimizing access inequities, can be associated with inappropriate requests for CT in the study setting. This is perceived as an unintended consequence that can be checked by the continuous sensitization and training of physicians, providing them with other cost-effective alternatives to CT as appropriate and encouraging the use of guidelines when uncertain. These measures, in our opinion, could help enhance the rational utilization of CT and reduce unnecessary exposure to ionizing radiation.

## Figures and Tables

**Table 1:** Demographic and socioprofessional characteristics of study participants.

Variables	Frequency (%)
Age group (years; *N* = 344)	
18–24	21 (5.7)
25–34	51 (13.7)
35–44	55 (14.8)
45–54	76 (20.4)
55–64	55 (14.8)
>65	114 (30.6)
Sex (*N* = 372)	
Female	167 (44.9)
Male	205 (55.1)
Marital status (*N* = 372)	
Married	232 (62.7)
Single	83 (22.3)
Widow (er)	42 (11.3)
Divorced	11 (3.0)
Living in union	4 (1.1)
Educational achievement (*N* = 347)	
<O level	158 (45.5)
O level or equivalent	61 (17.6)
A level or equivalent	64 (18.4)
Degree or equivalent	45 (13.0)
Master and above	19 (5.5)
Employment status (*N* = 294)	
Employed, with contract	82 (27.9)
Employed, with no contract	2 (0.7)
Self-employed	115 ( 39.1)
Unemployed	12 (4.1)
Retired	83 (28.2)
Socioeconomic status quintiles (*N* = 370) from the lowest (1) to the highest (5)	
SES quintile 1	74 (20.0)
SES quintile 2	75 (20.3)
SES quintile 3	75 (20.3)
SES quintile 4	79 (21.4)
SES quintile 5	67 (18.1)
Health insurance ownership (*N* = 370)	
Yes	38 (10.3)
No	332 (89.7)

**Table 2:** Health insurance types.

Insurance type	Frequency (%)
Assistance by a church	2 (5.26)
Private insurance company	5 (13.16)
Insurance provided by the employer	26 (68.42)
Local mutual health insurance scheme	5 (13.16)
Total	38 (100)

**Table 3:** Health insurance ownership and CT appropriateness.

	CT appropriateness, *n* (%; 95% CI)			Total, *n* (%)
	Category A^*∗*^	Category B	Category C	
No health insurance ownership	218 (69.4; 64.3 – 74.5)	74 (23.6; 18.9 – 28.3)	22 (7.0; 4.2 – 9.8)	314 (100)
Health insurance ownership	25 (65.8; 50.7 – 80.9)	6 (15.8; 4.2 – 27.4)	7 (18.4; 6.1 – 30.7)	38 (100)
Total	243 (69; 64.2 – 73.9)	80 (22.7; 18.3 – 27.1)	29 (8.2; 5.4 – 11.1)	352 (100)

^*∗*^According to the American College of Radiologists Appropriateness Criteria^®^, Category A indicates the requested imaging modality (CT in this case) is the most appropriate; Category B indicates the requested study could be appropriate, especially in the absence of any other reasonable alternative; Category C implies the requested imaging study is not appropriate for the clinical indication.

**Table 4:** Summary analysis of the relationship between CT request appropriateness and health insurance ownership.

	CT appropriateness, *n* (%; 95% CI)		Total, *n* (%)
	Category C^*∗*^	Categories A and B merged	
No health insurance ownership	22 (7.01; 4.2 – 9.8)	292 (92.99; 90.2 – 95.8)	314 (100)
Health insurance ownership	7 (18.42; 6.1 – 30.7)	31 (81.58; 69.3 – 93.9)	38 (100)
Total	29 (8.24; 5.4 – 11.1)	323 (91.76; 88.9 – 94.6)	352 (100)

*χ* ^2^ = 5.84; *p*=0.016. ^*∗*^Category C corresponds to inappropriate CT requests.

**Table 5:** Regression analysis of the appropriateness of CT requests.

Variables	Univariate analysis		Multivariate analysis	
	Crude OR (95% CI)	*p* value	Adjusted OR (95% CI)	*p* value
Age (years, *N* – 354)	1.00 (098 – 1.02)	0.827	0.98 (0.96 – 1.01)	0.222
Gender (*N* – 354)				
Female	1		1	
Male	0.53 (0.23 – 1.20)	0.129	0.72 (0.30 – 1.72)	0.461
Education (*N* – 288)				
Completed ordinary level or less	1		1	
Beyond ordinary level	0.42 (0.18 – 0.97)	0.043	0.35 (0.13 – 0.91)	0.032
Socioeconomic status (*N* – 352)	1.27 (0.67 – 2.38)	0.462	1.77 (0.92 – 3.43)	0.088
Health insurance (*N* – 352)				
Yes	0.33 (0.13 – 0.84)	0.020	0.37 (0.14 – 1.01)	0.053
No	1		1	
Healthcare provider (*N* – 344)				
Specialist doctors	0.79 (0.36 – 1.78)	0.578	0.77 (0.33 – 1.78)	0.540
General physicians	1		1	

Model *p* value – 0.062; *R*^2^ – 0.062; OR: odds ratio; CI: confidence interval.

## Data Availability

The dataset on which the findings of this study are based is available at https://doi.org/10.17632/r4dmt58v3r.1.
